# A New Method for Acoustic Priority Vehicle Detection Based on a Self-Powering Triboelectric Acoustic Sensor Suitable for Low-Power Wireless Sensor Networks

**DOI:** 10.3390/s21010158

**Published:** 2020-12-29

**Authors:** Quentin Quevy, Gianluca Cornetta, Abdellah Touhafi

**Affiliations:** 1 Department of Engineering Technology (INDI), Vrije Universiteit Brussel (VUB), 1050 Brussels, Belgium; Abdellah.Touhafi@vub.be; 2 Department of Information Engineering, San Pablo-CEU University, 28668 Madrid, Spain; gcornetta.eps@ceu.es

**Keywords:** energy harvesting, triboelectric generator, priority vehicle detection, acoustic energy harvesting

## Abstract

Traffic congestion is, on a daily basis, responsible for a significant amount of economic and social costs. One of the critical examples is the obstruction of priority vehicles during fast trajectories, which potentially costs lives and property in case of delay that is too great. By means of visual sensing methods, solutions and schedules have already been proposed for adjusting traffic light sequences depending on a priority vehicle’s position. However, these mechanisms are computation and power intensive. Deploying and powering a large-scale network will have a crucial economical cost. Furthermore, these devices will not always have access to sufficient power. To provide a solution, we developed an acoustic and self-powered device that can detect priority vehicles and can be cost effectively deployed to define a sensor network. The device combines the detection of priority vehicles and the harvesting of sound energy through triboelectrification. This paper will introduce the use of triboelectric energy harvesting, specifically in a self-powered wireless sensor network for priority vehicle detection. Furthermore, it shows how to increase the power performance of such a generator. Finally, the results are analyzed.

## 1. Introduction

The Internet of Things (IoT) has become an essential part of our lives. The total number of connected IoT devices is projected to rise to almost 76 billion worldwide by 2025, a fivefold increase in ten years [1]. Those devices are, in most cases, supplied by batteries. The use and replacement of batteries are often two of the most expensive aspects of IoT devices, and are also not environmentally friendly. The environmental impact of finding all the lithium required to enable enough energy storage has already become a severe issue in itself. Nevertheless, the impact caused by the extraction of lithium for the components of the Li-ion battery is less than 2.3%. The primary cause of the environmental burden is the supply of copper and aluminum [2]. Moving to recovery, recycling, and reuse of lithium battery materials is strongly encouraged. It has become clear that particular attention should be paid to the method of powering connected devices. Some solutions for increasing the lifetime of the battery are reducing power consumption, increasing energy efficiency, and extending the energy storage capacity.

Power management is one of the primary concerns in sensor networks. Some devices are powered by harvesting energy from the surrounding environment. Due to the size and price of the needed harvester, this option is often put away. A good compromise to overcome this issue is to combine the harvester with an energy storage element; for example, a battery. In such systems, the life of the battery is sometimes extended by up to a decade, even if only a small amount of energy is available in the environment. Such a combined powering structure shifts the need to have sufficient energy extracted from a source to the need to have a source present.

One of the energy sources that is the most omnipresent, but carries little energy, is sound. It is omnipresent, and even if it is not, in some sporadic cases, sound can even be produced from a distance. Sound is an essential factor in monitoring the activity of an environment. It helps to catalog wildlife, represent traffic, or even measure sound pollution. One of the main problems with such devices is that they require quite a large amount of power to supply microphones (mW/microphone) and to make the needed spectrum analysis. Therefore, harvesting energy from sound seems to be adequate, but challenging. The process used here to harvest energy out of sound is called triboelectrification, a process that uses electrostatic charges created on the surface of materials with different electron affinities. Since the creation of the first triboelectric nanogenerator in 2012, it is clear that a new way of harvesting energy was born. In 2016, the process was modeled in the book called “Triboelectric nanogenerators” [3], where every contact mode, equivalent circuit diagram, power characterization, and more were discussed. The author, Pr. Z.L. Wang, achieved a notable power density of 50 mW/cm${}^{2}$. This technology, which has enjoyed a growing interest, has inspired, through its structure and use, a lot of innovative works, such as: triboelectric and pyroelectric hybrid harvesters [4] and electrochromic energy harvesters [5]. Thanks to their properties, triboelectric devices are often used as sensors and harvesters at the same time [6].

Congestion severely affects our minds and costs billions of euros. Last year, for example, it cost over 300 billion dollars in the U.S. according to INRIX, a transportation consulting firm conducting large-scale research on global vehicular traffic. The U.S. experienced an increase in cost of 10 billion dollars since 2016. Our application concerns the mobility of priority vehicles, such as police cars or ambulances. Those kinds of vehicles need to move fast. They are slowed down due to traffic jams, traffic lights, and other various circumstances.

The solutions proposed to address this problem consist of measuring information like vehicle count, distance, and speed using visual sensing methods [7] or on-board transceivers [8]. The information is sent to a traffic management center to manage emergency traffic efficiently. A lot of processing power and speed are needed, but are not always available where monitoring is needed. If the system relies on vehicles equipped with transceivers, the vehicles that do not use such transceivers will not be detected or taken into account. To avoid these issues, we developed a device that can detect, thanks to a triboelectric sensor/harvester, different kinds of priority vehicles, and can send the data to, for example, a gateway or a traffic light in such a way that the traffic light can adapt its light scheme. The developed device produces its own energy and can be placed in a sensor network.

In this paper, we will propose and describe a novel self-powering priority vehicle detection device that relies on the acoustic emissions of the priority vehicle’s siren. In Section 2, we will explain the acoustic emission profile of the siren. Based on that emission profile, a dedicated sensor—harvester was designed, built, and tested. Then, an application scenario is discussed, in which multiple devices are deployed to form a sensor network. Section 3 gives a review of the test results.

## 2. Materials and Methods

### 2.1. Acoustic Emission Profile of Priority Vehicles

**Figure 1:**
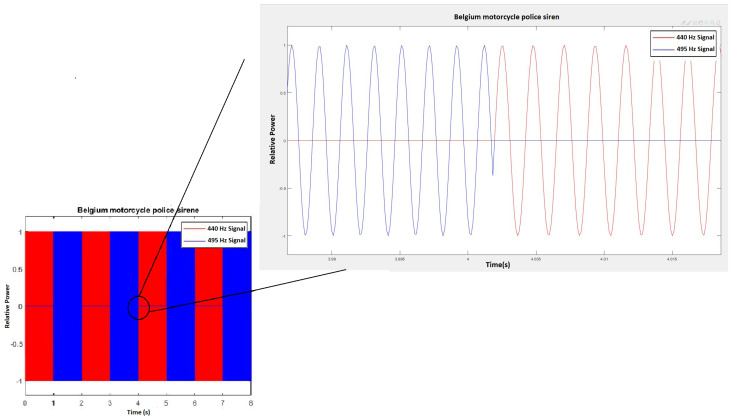
Relative power representation of a Belgian police motorcycle siren.

The main goal for this priority vehicle detection device (PVDD) is to detect and differentiate priority vehicles. First, what is going to be detected and how it is going to be detected are defined. The developed device was made specifically for the city of Brussels. The sirens of the public fire services and civil protection of the city of Brussels were the subject of a ministerial circular from 9 July 2013, which, among other things, limited the acceptable noise level to 95 dB (A) at night and at least 110 dB (A) during the day [9]. This circular also described the sound composition. The sirens consist of two tones of 440 and 495 Hz, alternating 20–30 times a minute, as can be observed in Figure 1. This concerns the medical, police, and fire vehicles.

The loudness of sound in decibels can be expressed as 10$log_{10}\frac{I}{I_{0}}$ with I being the intensity($\frac{P}{A}$) defined as power per unit area. ($I_{0}$ is equal to $10^{-12}$ W/m${}^{2}$ in order to normalize the logarithmic scale.) A 50 Watt speaker at 3 m of distance, for example, should have a loudness of 116 dB [10], which is very promising. In reality, due to a difference of impedance between the speaker and air, the efficiency is typically around $10^{-5}$, which lowers its loudness to 66 dB. Table 1 depicts some typical sound pressure levels of familiar sound sources.

#### 2.1.1. Sound Energy Harvesting

Now that the type of sound is settled, we need to know how to harvest energy from it. Several techniques can be used to harvest energy from sound. The most common are: piezoelectric, electromechanic, and triboelectric harvesters.

A piezoelectric element consists of two conductive plates, between which we put a non-conductive crystalline/ceramic plate. Mechanical stress applied to the piezoelectric element results in a difference in electric potential. The two electrodes collect and lead the charges to the output. Piezos show good performance in extracting energy from vibrations. The resulting voltage typically lies between 0 and 5 V. A voltage multiplier can be used at the rectifier stage to produce higher voltages [11]. In many use cases, several piezos have to be connected to achieve useable power performances, resulting in a wide array of piezos.

Like microphones or speakers, electromagnetic sound energy harvesters are based on Faraday’s law. Faraday’s law states that the rate of change of magnetic flux through an area enclosed by a loop is equal to the electric field’s magnitude around that closed loop. In other words, when a magnetic field is moving through a conductive closed loop, an electric current rises, or vice-versa. Such harvesters produce little energy and are hard to manufacture to fulfill our needs.

The last method for harvesting energy from sound is by using a triboelectric generator. The principle of triboelectrification can be explained as the effect that occurs when two dielectric materials with different affinities for charges (triboelectric series)—e.g., Kapton, a well-known polyimide and poly-methyl-methacrylate (PMMA)—enter into friction, and are brought into contact or separated from each other. Those materials brought into contact drive a difference in electric potential. Thanks to two electrodes put at the bottom and top of the generator, the electric charges created are collected and led to the output. Such generators can quickly be produced in every size and, as such, can be adapted to every application. Since the triboelectric process was first modeled [3], the first triboelectric acoustic energy harvesters were described. In 2015, an ultrathin copper, Teflon (PTFE) and paper-based harvester populated with micro-holes and carbon nanotubes was described [12]. The device is capable of delivering a maximum power density of 121 mW/m${}^{2}$. It has a relatively high power output, but comes with a high fabrication cost. In 2018, a triboelectric acoustic energy harvester was researched for high-speed railways [13]. The technique makes use of a Helmholtz resonator and a single electrode setup. Because it is a single-electrode method, the output power is low. To harvest enough energy to power a controller, multiple harvesters are placed in a matrix disposition. Our approach has a lower power output than, for example, the electrochromic nanogenerator based on Cu foam [5], but has a much lower cost. Nevertheless, as a low-cost acoustic energy harvester, it has a higher output power than the ones currently developed. Table 2 gives a short overview of the different methods’ output power.

#### 2.1.2. Increasing Triboelectric Performance

In order to harvest sufficient energy without using nanotechnology, we have to design our harvester to be as efficient as possible. This section describes the triboelectric effect and how to increase its performance.

**Figure 2:**
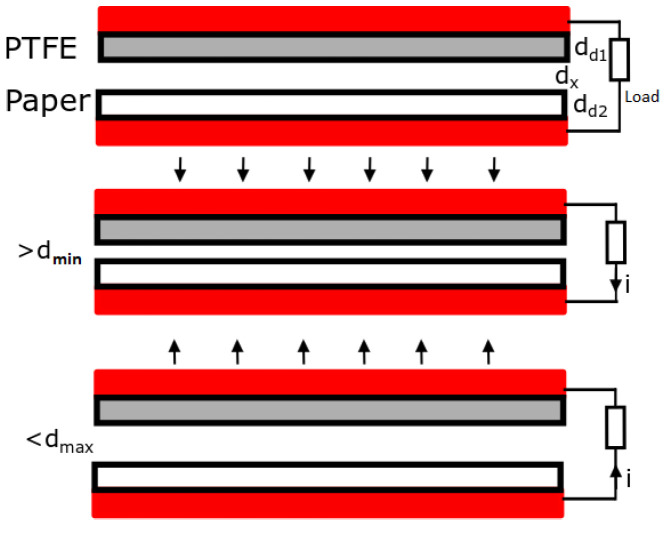
Triboelectric process.

As depicted in Figure 2, the triboelectric effect is a contact-induced electrification in which a material becomes electrically charged after it is brought in contact with a different material through friction [3]. The characteristics of the triboelectric generator, listed below, define the output power.

- Choice of material
- Contact gap distance ($d_{min}$ and $d_{max}$)
- Dielectric thickness ($d_{d1}$ and $d_{d2}$)
- Dielectric surface microstructure and hybridization
- Dielectric surface contact area
- Electrode
- Voltage superposition

Double- and single-dielectric attached-electrode contact-mode triboelectric generators are made using two conductors and one or two dielectric materials. The dielectric layers have tendencies to attract or repulse electrons. The charge transfer highly depends on the chosen materials. Table 3 shows some examples of charge transfer affinities of materials. For a sound-energy harvester’s membrane, a flexible but durable dielectric has to be chosen.

The intrinsic output characteristics of an attached contact-mode triboelectric nanogenerator (TENG) $Q_{sc}$ reach 90% of its final saturation value δS when x changes from 0 to 10$d_{d1/2}$ (with $d_{d1/2}$ being the effective thickness of the dielectrics). It is important that the minimum contact distance reaches as close to zero as possible. A minimum distance $d_{min}$ approximately equal to $10d_{d1/2}$ leads to no charge transfer. The charge transfer efficiency $\eta_{CT}$ is described by the following equation:

(1) $$\eta_{CT}=\frac{1}{1+\frac{C_{1}\left(x=x_{max}\right)}{C_{2}\left(x=x_{max}\right)}}-\frac{1}{1+\frac{C_{1}\left(x=0\right)}{C_{2}\left(x=x_{0}\right)}}$$

The equivalent circuit diagram is formed by two series capacitors, with $C_{1}$ and $C_{2}$ being the capacitance. The core mechanism of TENGs is that the change of the capacitance ratio $C_{1}$/$C_{2}$ induces electron transfer between the electrodes.

When $d_{min}$ is equal to or greater than $10d_{d1/2}$, $C_{1}$ becomes nearly 0. The $C_{1}$(x)/$C_{2}$(x) capacitance ratio of the theoretical model of a dielectric-to-dielectric attached-electrode parallel-plate contact-mode TENG will be close to 0, resulting in zero charge transfer [3]. Note that if the dielectrics never touch each other ($d_{min}>$ 0) and maintain a minimum distance, the output will be reduced, but the lifetime of the triboelectric generator will be extended [15].

The thickness of the dielectric layer plays a major role in two characteristics of the TENG. A thicker dielectric improves the surface charge density and increases the impedance of the TENG. Increasing the surface density enhances the output performance until the dielectric reaches a maximum value of stored charges. The excess thickness only increases the TENG’s impedance and will not further contribute to additional charge accumulation.

Output current and voltage are both proportional to the triboelectric charge density of the friction layer. Triboelectric charge density depends mainly on two factors: appropriate materials and the building surface microstructure. An enhancement of the charge density can be achieved by combining the advantages of different materials into one, creating a composite structure [16]. The added dielectric layer should have lower carrier mobility or lower intrinsic carrier density than the superposed dielectric layer [17]. The resulting composite material improves the total storage charge and storage depth of the layer.

To further improve the TENG, its nanostructure has to be adapted. This is often done by having a layer with nanotubes; for example, PTFE and another layer with nano-/micro-holes. The contact area will increase significantly, and thus, so will the output power.

Two electrodes collect the induced charges. The choice of an electrode depends on its conductivity. Therefore, copper showed the best results due to its high conductivity.

**Figure 3:**
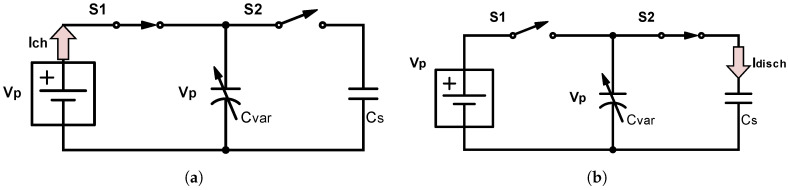
(**a**) Precharging the variable capacitor (C_Var_) (when C_Var_ = C_max_) and (**b**) discharging the C_Var_ (when C_Var_ = C_min_) to a storage capacitor (C${}_{s}$) [18].

The last method for increasing a TENG’s output performance is to add a small voltage source to the TENG. Figure 3 depicts the equivalent circuit, with the harvester being the variable capacitor.

**Figure 4:**
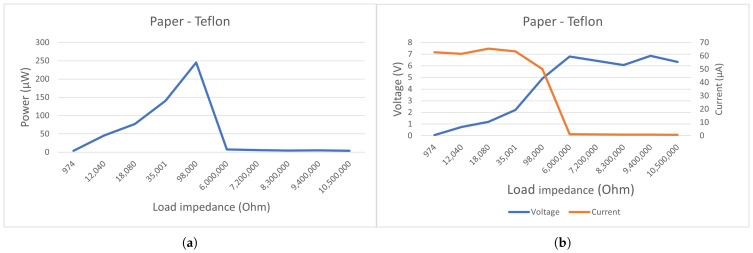
Electrical characterization of a triboelectric nanogenerator (TENG) with a resistive load. Converted power versus load resistance (**a**). The TENG’s peak current/voltage (**b**).

Figure 4 shows the output performances of our paper/teflon TENG under different load resistances. We can observe in Figure 4a that the optimal load resistance lies around 100 kΩ. In order to keep the load resistance low and increase the total power output, a voltage source can be placed in series with the generator. The low $V_{SC}$ contributes to the poor performance of the output. By adding a voltage source in series, we can benefit from the high $I_{SC}$ without reducing the power too much.

#### 2.1.3. Detection

The structure of the PVDD was adapted to detect priority vehicles as a function of the profile described in Table 4. A sound source (priority vehicle) is moving toward an observer (PVDD) at a maximum continuous speed of 150 km/h. We assumed that the traffic lights are placed in a rural environment where more than 150 km/h is rarely reached. The maximum distance from a traffic light is 2 m. This reproduces the test conditions for our PVDD, as described in Section 3.

Taking the doppler effect into account, our source undergoes a small frequency shift, as depicted in Table 4. With a velocity of 150 km/h and a total distance of four meters, the priority vehicle will be detected within at least 96 ms. The doppler equation is defined as follows:

(2) $$f^{\prime }=\frac{v\mp v_{0}}{v\mp v_{s}}\times f_{0},$$

assuming a 343 m/s sound velocity (*v*), 0 m/s observer velocity ($v_{o}$), and source velocity ($v_{s}$).

**Figure 5:**
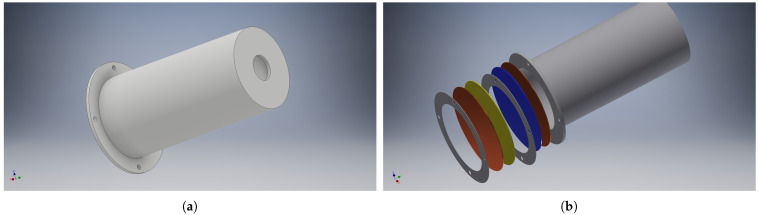
Neck-less Helmholtz resonator (**a**). Three-dimensional structure with dielectric and electrode layers and spacers (**b**).

The triboelectric harvester’s primary structure consists of a 3D-printed neck-less Helmholtz cavity, as depicted in Figure 5a. The size of the harvester will determine the resonant frequency of the system. For a neck-less Helmholtz harvester, Equation (3) can be derived for the resonant frequency $f_{h}$. Because it is more efficient for the given frequencies, it is easy to distinguish priority vehicles from others. The sensor can be used as a harvester or strictly as a sensor depending on the environment. For the second case, an additional power source or harvester, such as a small solar panel, provides the extra energy needed.

(3) $$\begin{array}{cc} f_{h} & =\frac{c}{2\pi }\sqrt{\frac{S}{VL_{eq}}}\\ \; & =\frac{c}{2\pi }\sqrt{\frac{S}{V(L+0.3D)}} \end{array}$$

Here, *c* is equal to the velocity of air; *S* stands for the surface area of the neck; *V* denotes the cavity volume; *L* stands for the length of the neck; and *D* stands for the neck’s diameter.

This results in a harvester with a diameter, D, of 5 cm and a height of 10 cm. The neck has a diameter of 1.2 cm and a height of 2 mm in order to resonate at a frequency of approximately 470 Hz. The exact frequency response is further discussed in Section 3.

#### 2.1.4. Triboelectric Generator

The triboelectric generator is placed on the top of the cavity. The first layer consists of a copper plate with a 12 μm layer of uncoated paper acting as the primary dielectric. This first layer is punched with acoustic holes of 400 μm. The second layer consists of a thin 40 μm copper layer and a 330 μm PTFE layer, which will act as the second dielectric. The copper side is attached to a polyethylene terephthalate (PET) film, which helps the second layer to have less friction during displacement. Barium titanate (BaTi03) is placed in between the PTFE and the copper layer. By incorporating high-dielectric-constant material (BaTi03), the composite will show a significant increase in permittivity and polarization, contributing to the increase in voltage, current, and charge density [15].

**Figure 6:**
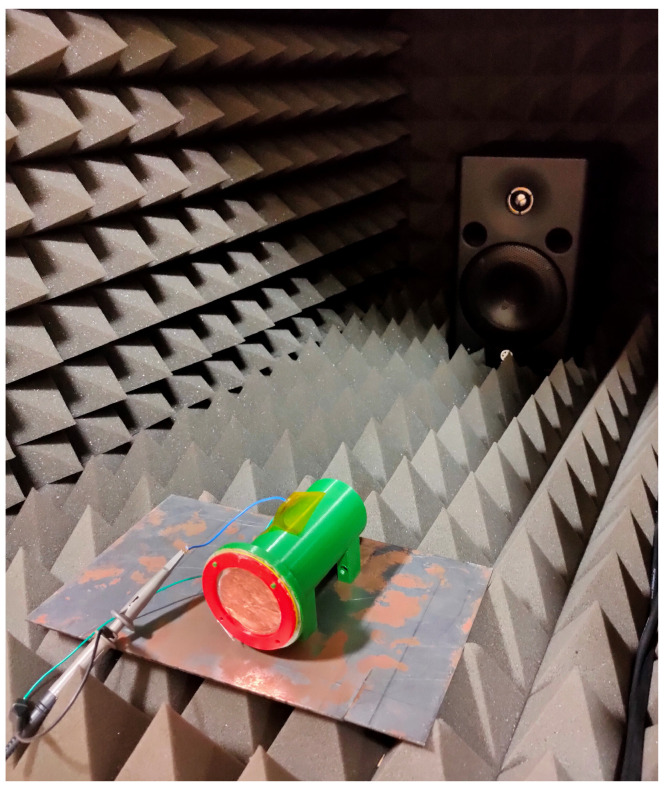
Triboelectric harvesters.

Figure 5 presents a 3D model of the harvester, and in Figure 6, a harvester is presented in the test setup inside an anechoic box [19]. The spacer can be observed due to its yellow color.

**Figure 7:**
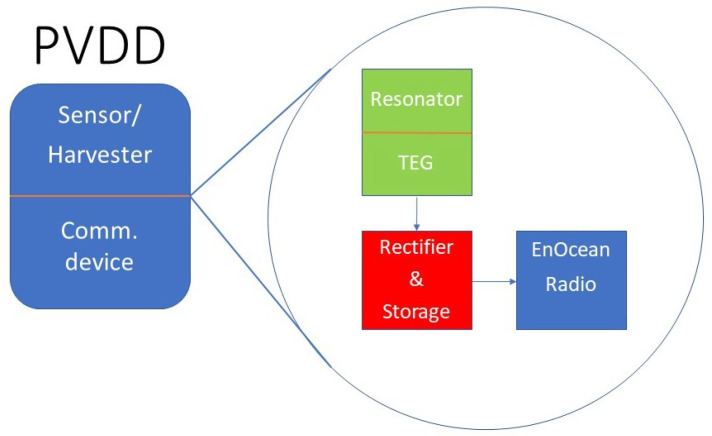
Priority vehicle detection device (PVDD) architecture.

**Figure 8:**
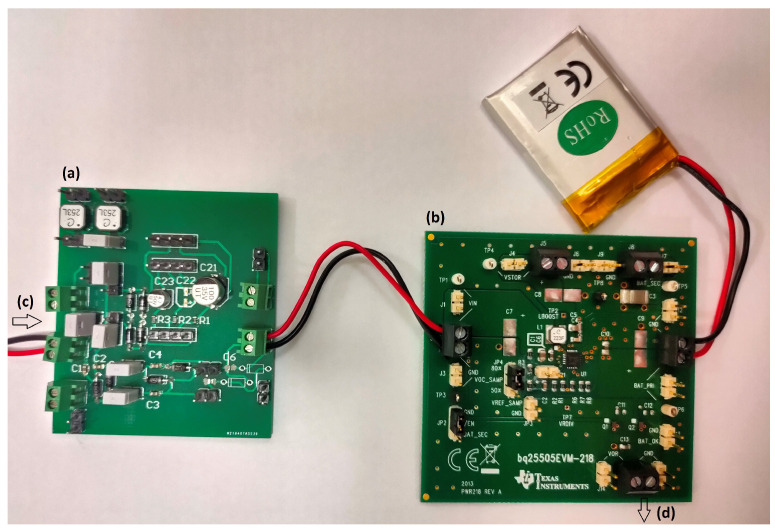
(**a**) Rectifier; (**b**) bq25505 battery controller evaluation board; (**c**) harvester’s output; (**d**) battery controller’s output.

Each node is composed of three common parts: a sensor, a power management unit, and an EnOcean communication node (Figure 7). The sensor consists of a resonator and a triboelectric generator. The power is rectified and put into a storage element (Figure 8a). If the communication node is a PTM200 the storage element is a capacitor, only the left printed circuit board (PCB) on Figure 8 is used. Otherwise, the bq2550 (Figure 8b), an ultra-low-power power management integrated circuit (IC) with a boost charger from Texas Instruments, charges a 3.7 V 600 mAh lithium-ion battery to power the circuit. Labels (c) and (d) in Figure 8 respectively point to the harvester’s output and the battery controller’s output.

### 2.2. Sensor Network

This section investigates if such a harvester could be used in a sensor network. First, different communication protocols were analyzed. Then, measurements of power requirements were taken. After this, the sensor network architecture is given and an application scenario is proposed. The actual deployment of this network is left for future work. Currently, network-specific power measurements are based on vendor-specific technical datasheets.

#### 2.2.1. Communication

Table 1 shows that sound contains a limited amount of energy. The energy required by the sensor node has to be limited to the restricted energy budget. EnOcean is a company for green and smart devices. In 2009, they developed wireless devices that can send messages by the force of a pushbutton switch. The PTM210, together with a USB300 device, enables the implementation of wireless remote controls without batteries. A built-in dynamic power generator provides enough power to transmit a message. The USB300 and the PTM210 [20] provide a bidirectional EnOcean radio protocol through a virtual serial interface. The device uses 0.1 mJ per operation (tx packet) with an efficiency of 60%. This amount of energy can be harvested with a minimalistic triboelectric device. By applying our harvester on their devices, a simple proof of concept has rapidly been created.

#### 2.2.2. Protocols

Four protocols were chosen to communicate with the nodes, with the main concern being the power consumption (Table 5). Firstly, there is the EnOcean protocol, an integrated and ready-to-go solution. Then, there is MiWi, a low-power protocol supporting peer-to-peer and star network topologies. Next, there is the Narrowband Internet of Things (NB-IoT), a radio technology created by the 3rd Generation Partnership Project (3GPP) to offer cellular services in the IoT. The last protocol is LoRa, a well-known long-range, low-power protocol.

Table 5 and Table 6 show a comparison of the previously mentioned communication protocols.

The EnOcean protocol was specially designed for energy harvesting of slight mechanical motion. The wireless standard was ratified according to the international standard ISO/IEC 14543-3-10, covering the Open Systems Interconnection layers 1–3, which are the physical, data link, and networking layers. The packets sent are small, making it hard to avoid collisions. However, reliability is guaranteed because each packet is randomly sent three times.

MiWi is a proprietary wireless protocol designed by Microchip Technology. MiWi uses small, low-power digital radios based on the IEEE 802.15.4 standard, and is designed for low-power, cost-constrained networks. It is similar to the Zigbee protocol, and can only be used with Microchip hardware devices, such as the SAMR30.

LoRa instead uses license-free sub-gigahertz frequency bands, like the 433 and 868 MHz bands, in Europe. LoRa enables long-range transmissions (more than 10 km in rural areas) with low power consumption. The technology covers the physical layer, while LoRaWAN, among others, covers the upper layers. LoRa induces duty-cycle restrictions depending on the region. In Europe, the duty cycle is limited to 1%. Because the messages in our application are very short, it will not form a bottleneck.

The NB-IoT protocol, released in 2015, is a communication protocol for Low-Power Wide-Area Networks (LPWAN) and was specially designed for the IoT. It relies on the existing 4G network and is 5G ready. Mainly in unused 200 kHz bands that were previously used for the global system for mobile communications (GSM), NB-IoT has increased system capacity and spectral efficiency. With a high modulation rate, it can exchange more data at a lower transmission rate. The main constraint of this protocol is that it has latencies of up to 10 s.

#### 2.2.3. Hardware

**Figure 9:**
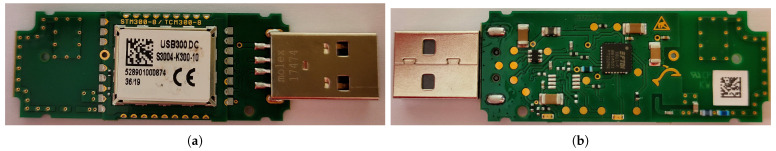
USB300.

The hardware used for the test setup consists of PTM200 and USB300 nodes from EnOcean and RN2483 motes from Microchip. The USB300 nodes are bidirectional communication devices that consist of a TCM310 radio chip Figure 9a and an FT232RQ UART to USB converter with some LEDs Figure 9b.

**Figure 10:**
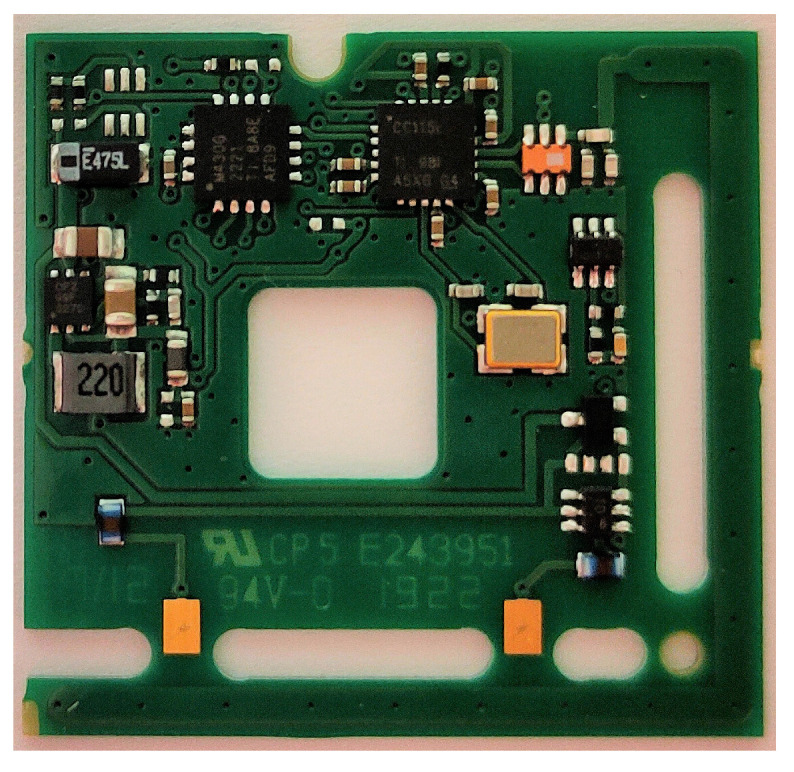
PTM200.

The PTM200 has an ultra-low power consumption, but has a unidirectional communication system; it can only send messages. Because of the unidirectional capability, both the PTM200 and the USB300 are used. The connection between the harvester and the node is made via the two copper pads shown in Figure 10. Due to the ultra-low energy requirements in the range of 100 μJ for the TCM310 and 1 μJ for the PTM200 (Table 7), the size of the harvester can be limited a lot. The proposed harvester’s volume equals approximately 196 cm${}^{3}$ (Figure 6).

The RN2483 is a fully certified (LoRa Alliance Certification) 433/868 MHz module based on wireless LoRa technology. The module is specially designed for ease of use, thanks to the incorporated microcontroller and the command interface, which shorten development time.

### 2.3. Harvester

#### Power Requirements

To size the harvester, we first investigated the power consumption of the communication devices. Table 7 illustrates the amount of energy measured when the nodes are sleeping, transmitting, or receiving a message. The measurements were done with an ina139 current monitor. The IC has an integrated amplifier that allows the measurement of smaller currents. For the transmission, the following equation was used:

(4) $$t=\frac{Packetsize(bits)}{rate_{tx}}$$

(5) $$E_{tx}=P_{tx}\times \;t$$

The other values could be derived in the same way as the transmission, except for the PTM210, whose values were taken from its datasheet. Due to the previously described downsides of the protocols, the MiWi and NB-IoT nodes were discarded.

All three modules apply to different use cases. The RN2483 is the most power-expensive device, but has the most extended range. This can be adequate for the gateway. The EnOcean family devices are ultra-low-power devices that can be used as transmission nodes.

### 2.4. System Architecture

**Figure 11:**
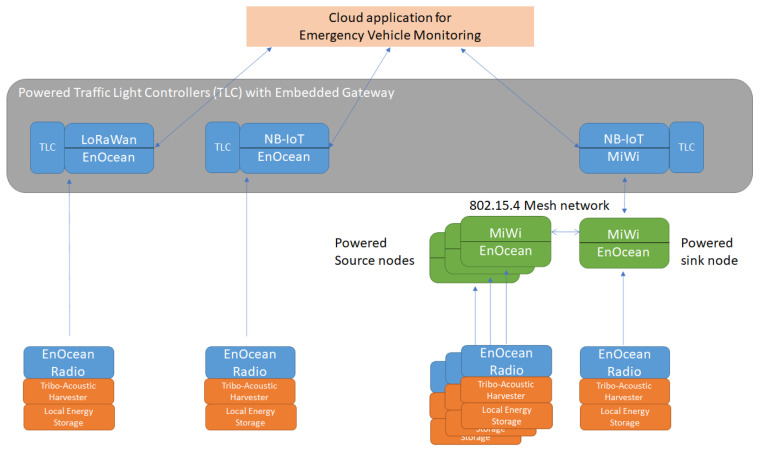
Sensor network architecture.

Two topologies are considered. The first one is when the gateway communicates directly with bus networks made of PVDDs (EnOcean). The second scenario is when a meshed network is created using MiWi motes. The final node in the network is the gateway, which will communicate with a cloud application. As depicted in Figure 11, the gateway can communicate via LoRa/NB-IoT and EnOcean/Miwi.

The application scenario consists of a priority vehicle that will pass by several detection devices. These nodes wake up and forward a message to a communication node settled on the next traffic light. This message is sent further until it reaches the gateway to trace the trajectory of the priority vehicle. A message can be discarded for three reasons: The source does not comply with the profile (Table 4) determined previously, or the priority vehicle stops its sirens, and thus, no other signal is acquired by the next PVDD. The last rule is that the detection has to occur at least ten times in a 96 ms time interval in order to be validated. This process is continuously repeated while the priority vehicle passes by. In the meantime, traffic lights can be adapted in real time.

**Figure 12:**
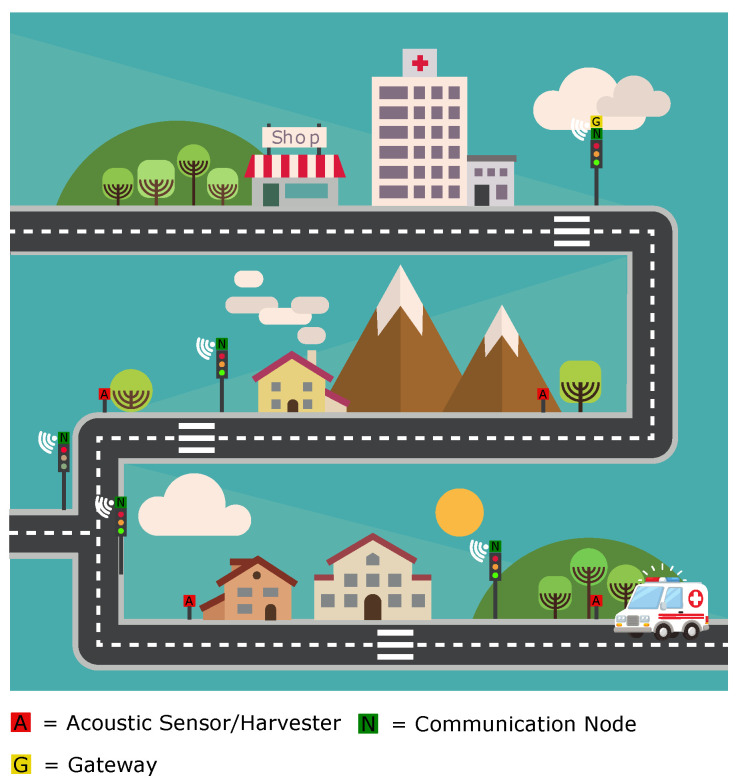
Application scenario (background image: Freepik.com).

The gateway node includes LoRa and will pass the information to a central computing system, which is informed of the new traffic-light scheme. As depicted in Figure 12, the acoustic sensor/harvester nodes are composed of a PTM210 and the harvester/sensor, which detects the priority vehicle and sends the message to a communication node. The communication node consists of a TCM310 module.

## 3. Results

**Figure 13:**
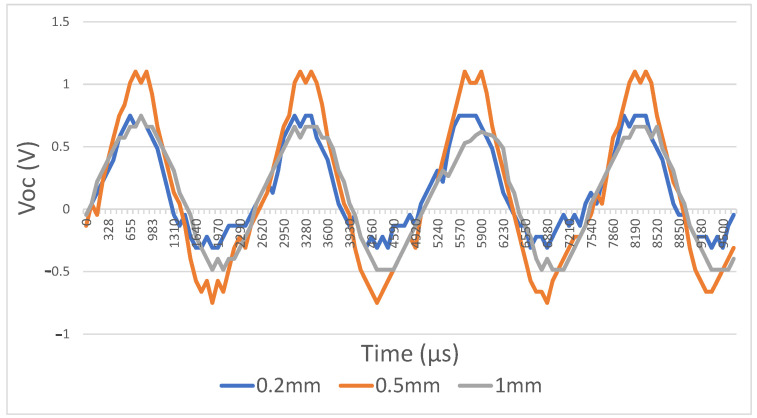
Open circuit voltage as a function of different maximum gap distances.

A speaker was placed two meters away from the device to trigger the harvester at a frequency of 495 Hz at 95 dB (measurement instruments used: MSOX3012A mixed-signal oscilloscope, INA139 current sensor, UMIK-2 minidsp measurement microphone, and Fluke 175 multimeter). Firstly, the dielectric layers were made from woven steel on one side and teflon on the other side. The first parameter we tested was the maximum contact gap distance. Three different-sized spacers (200 μm, 500 μm, and 1 mm) were printed. The best results were obtained at a distance of 500 μm (Table 8), Figure 13. One-millimeter spacing performed more poorly because it was equal to approximately seven times the effective thickness of the dielectrics, which is near the limit, resulting in zero power output. The 200 μm spacing performed more poorly because it induced little possibility for movement.

Next, we tested techniques to enhance the power output by combining different materials’ advantages. Here, carbon nanotubes were added between the dielectric layer and the electrode to lower the contact resistance. Another test was also performed by introducing barium titanate between those layers. Barium titanate is an oxide, and thus has a highly positive triboelectric affinity.

**Figure 14:**
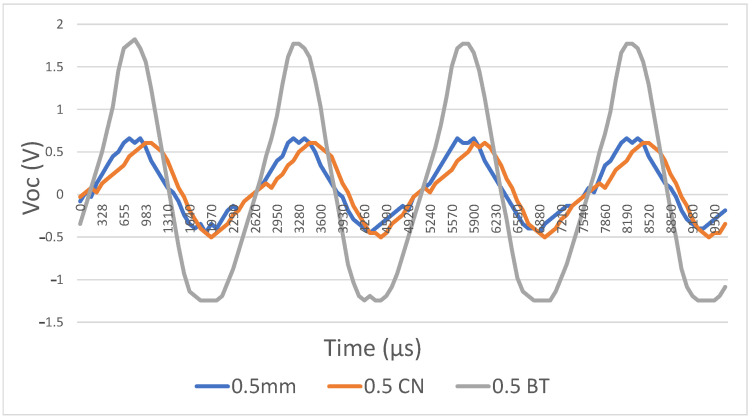
Open circuit voltage for different output power enhancement techniques.

Carbon nanotubes have better carrier mobility and higher intrinsic carrier density than the superposed dielectric layer, resulting in worse outputs (Figure 14). On the other hand, barium titanate showed very promising improvements by increasing the layer’s total storage charge, reaching a peak-to-peak voltage of 3.1 V.

**Figure 15:**
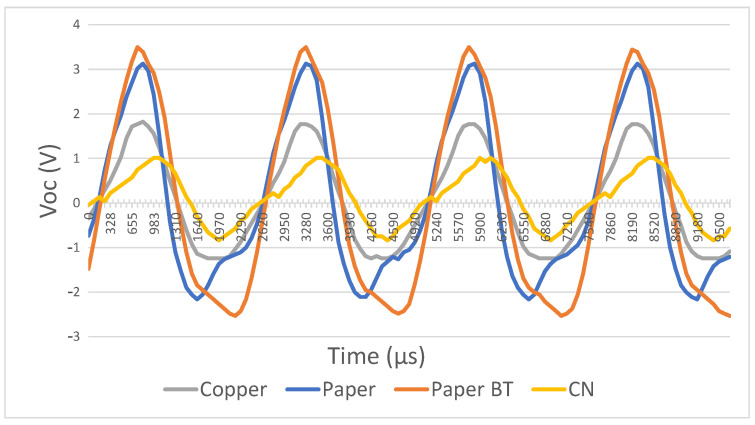
Open circuit voltage for different dielectric materials.

For the last test on the harvester structure, we adapted the material of the dielectric layers. Table 8 depicts the results of those tests. We achieved the highest output voltage with paper and teflon as the dielectric layer. We further improved the TENG by adding barium titanate between the electrode and the paper layer. The results are shown in Figure 15.

**Figure 16:**
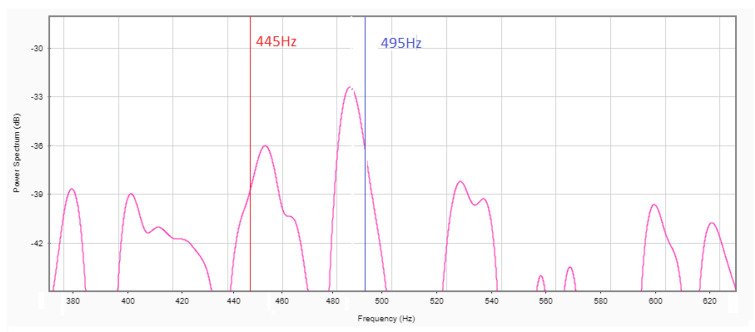
Frequency response of the harvester.

**Figure 17:**
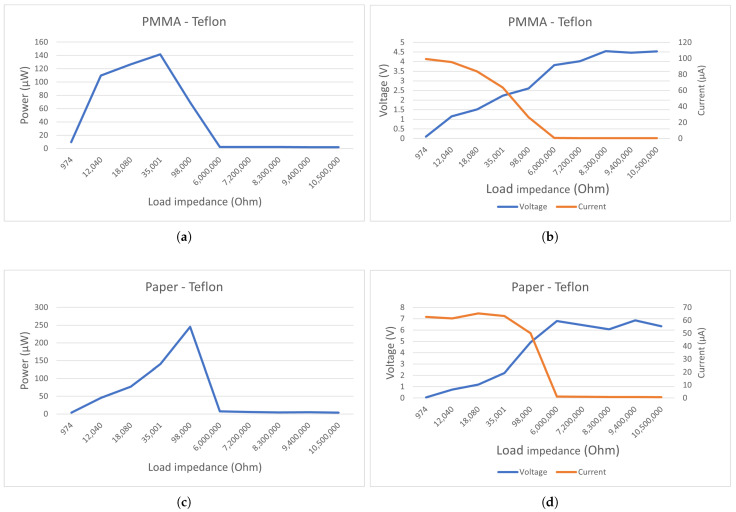
Converted power versus load resistance from PMMA-Teflon (**a**); Output current/voltage for different loads from PMMA-Teflon (**b**); Converted power versus load resistance from Paper-Teflon (**c**); Output current/voltage for different loads from PMMA-Teflon (**d**).

Now that the structure of the harvester/sensor is settled, we can test its frequency response. Figure 16 shows that the response is mainly centered around 490 Hz. The response lies ideally for the 495 and 445 Hz signals of a priority vehicle. The other peaks are, respectively, 6 and 3 db lower, which makes the identification of a priority vehicle simple. With this setup, the highest power output we found was equal to 245 μW at a voltage of 4.9 V. We did the same test with PMMA as a dielectric layer, but it performed less well (Figure 17) with 141 μW at a voltage of 2.2 V. From Figure 17a,b we can deduct that the best matching load impedance for this setup using paper lies around 100 kΩ, and using PMMA Figure 17c,d, it lies around 35 kΩ.

With the presented method, sufficient energy is produced to power the nodes. Table 9 shows how long it takes for a particular node to have enough energy to receive or transmit a message.

The most extended period to needed to harvest energy is approximately four seconds for the RN2483. The (traffic) light will power the node that is placed on it. The other nodes can store enough energy while a priority vehicle passes by to transmit and receive many messages.

## 4. Conclusions

Priority vehicles, such as police cars or ambulances, regularly need to move fast. They are often slowed down, resulting in severe costs. We developed a device that can detect different kinds of priority vehicles and send the data to control traffic lights. The device containing our 3D-printed harvester dedicated to harvesting a 445/495 Hz mixed source can detect acoustic energy at the same time. Our device has a high output performance (245 μW) compared to other low-cost acoustic solutions, is self-powering, and is small (no array of harvesters needed). Furthermore, we also presented several network architecture possibilities that integrate a self-powered tribo-acoustic harvester and sensor as part of the priority vehicle detection system. In all cases, the self-powered sensor uses the EnOcean communication protocol to communicate directly with a powered gateway. Two different interconnection schemes can be adopted. The first interconnection scheme connects the EnOcean radio with a smart traffic light controller, which includes an embedded gateway that supports a full-stack IoT protocol (NB-IoT or LoRaWan) and an EnOcean Radio. The second connection scheme provides a two-layered interconnection scheme. The first layer connects the self-powered sensors with a powered mesh network. The mesh network routes all the data to a sink, from which the data are sent further to a smart traffic light controller.

The next steps in our research will consist of the deployment of the PVDDs proposed in this work in outdoor scenarios for long-term testing. Improvements to the PVDDs that are based on simulations and measured performance results will be worked out.

## Figures and Tables

**Table 1:** Sound pressure level and sound power of common sound sources.

Sound Energy Source	Distance	Sound Pressure Level	Sound Power
Car	2 m	80 dB	0.1 mW
Car	10 m	75 dB	32 μW
Train	2 m	86 dB	0.4 mW
Bus	5 m	82 dB	0.16 mW
Running tap	1 m	65 dB	3 μW
Street with no traffic	/	60 dB	1 μW
Quiet room	/	30 dB	1 nW

**Table 2:** A Helmholtz resonator with a piezoelectric backplate mechanism (PZT 1), a Sol/Gel enhanced piezoelectric element (PZT 2), microelectromechanical systems (MEMSs), an electrochromic nanogenerator based on Cu foam (Tribo 1), and our harvester (Tribo 2).

	PZT 1 [14]	PZT 2 [14]	MEMS [14]	Tribo 1 [5]	Tribo 2 [This]
Power density (μW/cm${}^{2}$)	0.002 @110 dB	0.01 @100 dB	0.34 @149 dB	1300 @110 dB	3.1 @95 dB

**Table 3:** Materials’ characteristics.

Material	Affinity (nC/J)	Thickness (μm)
Copper	−5	40
Uncoated paper	+10	12
PTFE	−190	300

**Table 4:** Profile of sound source.

	$f_{1}$ ($f_{s}$ = 440 Hz)	$f_{2}$ ($f_{s}$ = 495 Hz)	Velocity	Period
Priority vehicle	[395 Hz, 495 Hz]	[440 Hz, 560 Hz]	41.67 m/s	96 ms

**Table 5:** Communication protocol comparison 1.

	Standard	Band	Topology	Downside
EnOcean	ISO/IEC 14543-3-10	868.3 MHz (USB300, PTM210)	Star	Only within EnOcean framework, collision avoidance/no detection
MiWi	IEEE 802.15.4	2.4 GHz	Mesh	Proprietary
NB-IoT	3GPP R13	800/900/1800 MHz (EU)	Star	Latency, data plan needed
LoRa	Private LoRa Network	433/868 MHz (EU)	Star	Low transmission rate, collision avoidance/no detection

**Table 6:** Communication protocol comparison 2.

	Range	Packet Size	Trans. Rate
EnOcean	30–300 m	14 bytes	125 kbits/s
MiWi	20–100 m	127 bytes	256 kbits/s
NB-IoT	<35 km	32 bytes	200 kbits/s
LoRa	5–15 km	51–255 bytes	27 kbits/s

**Table 7:** Summary of the power consumption of different nodes. * LoRa technology, ** EnOcean technology.

	Sleep (μW)	Transmit (μJ)	Receive (μJ)	Trans. Bit (μJ)	Receiv. Bit (μJ)	Parameters (TX)
RN2483 *	5.94	458.1	1028.5	0.25	0.35	+5.0 dB m, CS7, 255 bytes
PTM210 **	/	0.1	/	0.001	/	+5.0 dBm, 14 bytes
TCM310 **	0.99	70.1	97.6	0.63	0.87	+5.0 dBm, 14 bytes

**Table 8:** Output voltage for different spacings and materials.

Open Collector Voltage for Different Materials		
Dielectric	d_max (mm)	Voc (V)
Woven steel	0.2	1.23
	0.5	1.85
	1	1.23
Copper	0.5	3.12
Aluminum	0.5	4.48
Paper	0.5	5.34

**Table 9:** Amount of time until enough energy is harvested.

	Tx RN2483	Rx RN2483	Tx PTM210	Tx TCM310	Rx TCM310
Time (s)	1.87	4.19	0.4 μ	256 μ	356 μ

## Data Availability

The data presented in this study are available on request from the corresponding author.
